# Is premature theorizing hurting skill acquisition research?

**DOI:** 10.3389/fspor.2023.1185734

**Published:** 2023-10-10

**Authors:** Rajiv Ranganathan, Andrew Driska

**Affiliations:** Department of Kinesiology, Michigan State University, East Lansing, MI, United States

**Keywords:** theory testing, ecological validity, model tasks, sport coaching, information processing, ecological approach

## Introduction

*“…the dominant model of science in the field [that prioritizes experiments and hypothesis testing over real-world description] is appropriate only for a well-developed science, in which basic, real-world phenomena have been identified, important invariances in these phenomena have been documented, and appropriate model systems that capture the essence of these phenomena have been developed”. [Rozin, 2001, p. 2]*

Debates about competing approaches to skill acquisition (specifically information-processing vs. ecological based approaches) have dominated recent conversations, both among academics and practitioners. Our central argument in this article is that the above referenced quote by Rozin (1), originally made in the context of social psychology, is equally applicable to the field of skill acquisition in sport. Focusing on the current state of empirical work, we argue that there is not sufficient empirical data to constrain theories in skill acquisition research, and that trying to choose theories based on such limited data is both premature and detrimental to the development of the field itself.

### What data constrains theories of skill acquisition?

A theory is only as good as the data it explains. For example, consider the difference between skill acquisition and a closely related research field like motor control. In motor control, there are examples of well-established invariances such as Fitts' law (2, 3) or spatiotemporal characteristics of reaching trajectories (4, 5). These robust and replicable phenomena provide such a strong empirical constraint that any new proposed theory of motor control is a non-starter if it did not account for these fundamental observations (6, 7). In stark contrast, it is difficult to think of any finding that poses such a constraint to a theory of skill acquisition in sport. Instead, most phenomena in skill acquisition are characterized by two features—(a) they tend to be highly context-sensitive (i.e., influenced by factors such as the type of task and the stage of learning), and (b) they tend not to have quantitative process-level descriptions and instead focus mainly on outcome measures. While context-sensitivity is perhaps a reflection of the fact that skill acquisition is inherently sensitive to the learner and the learning context as seen by concepts such as desirable difficulties (8) or the challenge point (9), this also means that literature is filled with fragmented and seemingly contradictory findings. Coupled with the lack of quantitative descriptions, this has meant that current theories of skill acquisition tend to live within little bubbles of data.

## Can we really choose between theories of skill acquisition?

However, despite the lack of robust findings, there is an attempt to follow the conventions of more mature sciences such as doing “strong inference” (10). Theories are often posed as being diametrically opposite on particular issues (e.g., “in theory A, variability is good for learning, whereas in theory B variability is bad for learning”). But while this contrast is helpful in that it raises awareness about the different ways in which we think about these phenomena, in most cases, theories are not specific enough to make predictions. In other words, there is no subsequent critical experiment which could distinguish between theory A or theory B because the question itself—i.e., “is variability good or bad for learning”—is ill-posed without knowledge of the context (e.g., the stage of learning, how much variability is being introduced, and what type of task is being learned) (11). In these cases, the strategy of having theories compete by constructing these binary oppositions is less likely to advance science (12).

Another major issue regarding the data is the question of how much these results matter in real-world conditions. For example, most motor learning experiments, often used as the basis for skill acquisition, still rely on constrained tasks that rarely resemble the complexity of real-world (13, 14). In addition, with limited sample sizes being an important factor constraining research studies, it is ideal in an experimental sense to maximize the effect size (i.e., the potential difference between groups) as much as possible. As a result, the contrast between groups is often exaggerated with “strawman” versions of groups that bear little resemblance to real-world skill acquisition (15). For example, the information processing approach has been associated with “prescription” of an ideal movement pattern (often borrowed straight from a manual) with no room for individual differences, variability, or real-time flexibility, whereas the ecological approach has been associated with a trial-and-error “self-organization” approach to finding a movement solution with no room for planning, instructions demonstrations, or explicit strategies. Under these circumstances, it is easy to see how, depending on the experimenter's theoretical view, one could design an experiment in a context that makes one theoretical view look better than the other. As a result, even when these methods are directly compared (16–18), many researchers and practitioners remain unconvinced about the impact of such evidence on real-world contexts.

## Discussion

We wish to emphasize that our goal is not to criticize theorizing itself. Challenging the theoretical status quo has brought important new perspectives to the field, which in turn has guided empirical data collection in new directions. For example, the focus on organism-task-environment as a whole (19) is an important perspective change on the role of the coach in terms of being “environment designers” (20, 21). However, in prematurely trying to choose between theories, or derive implications for real-world situations, there is a danger of overgeneralization based on phenomena that have mainly been observed in niche experimental paradigms. We suggest two recommendations for improving the discourse- from a researcher's view and a practitioner's view.

### Theory building with real-world constraints

From a researcher's view, we propose that instead of the standard hypothesis testing/falsification paradigm, skill acquisition is much more suited to the “inductive theory building” approach (22), which argues for building a “substantial body of data” across different real-world contexts (using different methods, participants, time spans etc.). In particular, there is a need for data collection outside of the domain of lab experiments, which tend to focus on extremely time-limited constrained observations, that by themselves are too rudimentary for theory building. The need for field-based data is a not new observation (1, 23, 24) but the continued lack of field-based data in guiding theories of skill acquisition seems to point to a systemic problem in what type of research is incentivized, and the need for large scale collaborations (including adversarial collaborations). In short, we need phenomena that everyone can agree on before we can test theories that people may disagree on.

For an illustrative example of such theory building, one might look at the development of Self-Determination Theory (SDT) over the past 50 years. SDT began with experimental designs to examine the effects of incentives on creative problem solving using SOMA puzzles (25). When these experiments showed that incentives undermined participants' willingness to engage with the puzzles during a break period, it questioned behaviorist tenets of the importance of incentives and reinforcement in volitional behavior and gave rise to the core construct of *intrinsic motivation*. Today, SDT is a “meta-theory” composed of six subtheories, each with an explanatory capacity for specific aspects of volitional behavior. Two features of SDT stand out as a model for skill acquisition. First, although SDT enjoys rather wide acceptance amongst behavioral scientists; few would argue that SDT accounts for all variance in volitional behavior, and few would neglect the effects of reinforcement, group norms, or other psychosocial factors in certain contexts. Second, SDT emerged from tightly-controlled, laboratory-based experimental designs, but then expanded to less-controlled, field-based quasi-experimental and even descriptive and qualitative designs. This expansion required scientists to sacrifice internal validity (afforded by experimental designs) for increased ecological validity (afforded by field-based research), which in turn allowed the theory to have an impact on a wide range of fields (26).

### Adopting a wider lens when critiquing coaching practices

From a practitioner's view, we propose that skill acquisition and the utility of different coaching practices be examined from multiple lenses. Often, there is a tacit assumption that evidence-based coaching requires complete alignment of the goals of the researcher and the coach. However, this assumption can be misleading since the objectives of the researcher and the coach are quite different—researchers prioritize the search for systematic and generalizable principles whereas coaches are pragmatic and solution-focused. Recognizing this difference is an important aspect of the debate over skill acquisition and coaching practices and highlights the issue of why it may sometimes be non-trivial to translate research from controlled environments directly on to the field.

One example of how this difference manifests in practice is that a less optimal method of practice at one level may be preferred if it can be more efficient at a different level of analysis. For example, experimental comparisons of skill acquisition methods (such as blocked vs. random practice or constant vs. variable practice) assume that participants receive the same number of practice repetitions in each method. However, this assumption may not always hold in the real-world. With a fixed amount of practice time (which is typically the resource constraint), participants may often be able to do many more repetitions in a blocked or constant practice schedules because they require less changes in the environment and lesser effort from the coach. Similarly, certain types of isolated practice such as drills have been criticized because they remove the learner from the context. However, drills allow the coach to monitor several individuals at the same time. Therefore, as long as there is a non-zero learning benefit, some activities that seem suboptimal at one level (say the amount of learning/unit practice repetition) may actually be more efficient in terms of other levels (the amount of learning per unit time or per unit person-hours of coaching). Understanding these trade-offs at multiple scales of analysis is currently outside the domain of most experiments and highlights the need for more field-based work to complement lab-based work.

In addition, the solution-focused approach of coaches may also explain certain coaching practices. For example, many coaches still use an “ideal” movement pattern (e.g., that of an elite athlete) even if they do not believe in imprinting this movement pattern on the learner simply because there is no other alternative. Finding what the optimal pattern for a given individual is challenging even in the simplest of tasks because we currently do not have the framework to incorporate an individual's prior movement repertoire and preferences [cf. “intrinsic dynamics” (27)] into models of motor performance (28). Relying purely on discovery learning may be time-consuming and increase the risk of getting stuck in maladaptive movement patterns. A researcher faced with this problem has the option of choosing a different context that is more tractable for study, but this is not an option for the solution-focused coach. Therefore, using the elite athlete's movement patterns may represent a reasonable compromise in this scenario, as long as it is followed by a trial-and-error process to identify an individual's optimal solution. In addition, many pedagogical techniques may also be effective in that they satisfy different goals beyond skill improvement (29). For example, demonstrations are associated with increasing self-efficacy, reducing anxiety, and learning strategies or game plans (30). Although the above arguments are not meant as a general defense of all current coaching practices, it may be more fruitful to move debates away from “is coaching practice X important?” to “when is coaching practice X important?”. Such context-specific answers may be unsatisfying to many, but may be a necessary precursor to a unifying theory.

In summary, we need a large and robust body of data to advance theoretical debates on skill acquisition in a meaningful way. One approach that may especially be fruitful in this process is the “informed curiosity approach” (1). Taking a middle path between the two extremes of hypothesis testing and simply amassing more data, the informed curiosity approach is characterized by attempts to answer open-ended questions that prioritize description of phenomena in ecologically valid contexts. For example, to understand the role of variability in learning, instead of a typical hypothesis-testing approach that compares two groups, an informed-curiosity approach would focus on describing the entire dose-response curve between variability and learning using multiple groups in a real-world task. Such descriptions of a functional relation between variables (31) provides a much better constraint on theory development than the two-group design where the nature of the result (both in terms of direction and effect size) is often highly sensitive to how the two groups are selected (32).

Given the much higher effort involved in collecting this type of descriptive data and the breadth of skill acquisition in sport, a first step is to identify a few representative contexts that can be the focus of immediate efforts. Even in the laboratory setting, the use of select “model tasks” has been proposed to reduce task fragmentation and strike a balance between internal and ecological validity (32). By identifying a small set of common tasks that can capture different aspects of skill acquisition (similar to how model organisms are used in biology), researchers will be able to compile data across labs and obtain larger sample sizes, which can potentially lead to the discovery of invariances (in the same mold as Fitts' law) that become the basis for theorizing. However, based on similar efforts in other domains (33–36), achieving even this first step requires coordinated large-scale collaborations between academic researchers, sport scientists, coaches, and athletes in a way that runs counter to the current model of conducting research within a single lab. Creating the infrastructure and the incentive structure for these types of collaborations may ultimately be the most important piece for a theory of skill acquisition.
